# Typhoid Fever

**Published:** 1888-11

**Authors:** 


**Typhoid Fever.**—The Paris correspondent to *Philadelphia Medical Times* writes that intestinal antisepsis in typhoid-fever cases is receiving marked attention. In the treatment of such cases, Professor Bouchard first introduced naphthol; and Dr. Legroux is at present applying it in his service at the Children's Hospital with great success. As soon as a child is brought in suffering with symptoms of typhoid, the intestines are at once cleared out with calomel, given in doses of .30 to .60 centigrams, depending on the child's age. The next day the intestinal antiseptic treatment is commenced as follows: ℞.—Naphthol beta, bismuth salicylate, aa 2.50 grams. M. Divide into ten powders and give one every hour in a wafer, or mixed with a little milk or brandy. If the diarrhœa is not important, the bismuth may be left out and only the naphthol given; and if, on the contrary, there is constipation, then give the following: ℞.—Naphthol beta, 2.50 grams; magnesia salicylate, 2.50 to 5 grams. M. Divide into ten powders, and give one every hour until the bowels are free, and then continue with naphthol only, as before. There will be found a diminution or entire suppression of intestinal meteorism, and that the stools are disinfected, no longer give the fetid smell of typhoid. The mouth and tongue will clear up, the general condition will improve, and the disease will run a short course.

